# Continuous Renal Replacement Therapy for Acute Renal Failure in Patients with Cancer: A Well-Tolerated Adjunct Treatment

**DOI:** 10.3389/fmed.2016.00033

**Published:** 2016-08-03

**Authors:** Rebecca Fischler, Anne-Pascale Meert, Jean-Paul Sculier, Thierry Berghmans

**Affiliations:** ^1^Department of Intensive Care and Oncological Emergencies and Thoracic Oncology, Institut Jules Bordet, Université Libre de Bruxelles, Brussels, Belgium

**Keywords:** intensive care, neoplasms, acute kidney injury, renal dialysis, prognosis

## Abstract

**Introduction:**

Acute renal failure (ARF) has a poor prognosis in patients with cancer requiring intensive care unit (ICU) admission. Our aim is finding prognostic factors for hospital mortality in patients with cancer with ARF requiring renal replacement therapy (RRT).

**Methods:**

In this retrospective study, all patients with cancer with ARF treated with continuous venovenous filtration (CVVHDF) in the ICU of the Institut Jules Bordet, between January 1, 2003 and December 31, 2012, were included.

**Results:**

One hundred and three patients are assessed: men/women 69/34, median age 62 years, solid/hematologic tumors 68/35, median SAPS II 56. Mortality rate was 63%. Seven patients required chronic renal dialysis. After multivariate analysis, two variables were statistically associated with hospital mortality: more than one organ failure (including kidney) (OR 5.918; 95% CI 2.184–16.038; *p* < 0.001) and low albumin level (OR 3.341; 95% CI 1.229–9.077; *p* = 0.02). Only minor complications related to CVVHDF have been documented.

**Conclusion:**

Despite the poor prognosis associated with ARF, CVVHDF is an effective and tolerable renal replacement technique in patients with cancer admitted to the ICU. Multiple organ failure and hypoalbuminemia, two independent prognostic factors for hospital mortality have to be considered when deciding for introducing RRT.

## Introduction

Acute renal failure (ARF) is a frequent complication in patients with cancer. It may develop due to the cancer itself (multiple myeloma, neoplastic urinary tract obstruction, renal malignant infiltration), its treatment (nephrotoxic chemotherapy, tumor lysis syndrome …), or secondary complication (sepsis, nephrotoxic antibiotics, iodine contrast administration …) (1, 2). Up to 54% of patients with cancer admitted to the intensive care unit (ICU) develop ARF and some require renal replacement therapy (RRT) (3–5). Despite improvements in the general management of ICU patients with cancer, ARF worsens the prognosis. In patient with cancer requiring dialysis, hospital mortality rates are reported between 51 and 90% (6–8).

A few published studies are dealing with outcome of patients with cancer with ARF, who required RRT. Results are difficult to compare for several reasons as types and indications of RRT largely varying from one study to another or populations are not comparable. For example, the proportion of solid and hematologic tumors and the number of hematological transplantation were inconstant across the studies while these patients are carrying very distinct prognosis (7–14).

In a first study performed at the Bordet Institute, continuous venovenous hemodiafiltration (CVVHDF) was shown effective for the treatment of ARF in patients with cancer, including solid and hematologic tumors as well as auto- and allograft recipients (15). In the latter small retrospective study, the single independent poor prognostic factor for hospital mortality was the increasing number of organ failures. In another study with a similar case-mix, other authors confirmed the prognostic role of the number of organ failures on hospital mortality (16). As dialysis techniques evolve with time, we aimed at confirming our previously published results on the effectiveness of RRT in a larger cohort of patients with cancer. The primary objective of this retrospective study was the search of prognostic factors for hospital mortality in this group of patients that could eventually help in guiding decision of RRT.

## Patients and Methods

All consecutive patients admitted in the medical ICU of the Institut Jules Bordet between January 1, 2003 and December 31, 2012 were recorded. The records of the patients with ARF requiring continuous venovenous RRT at admission or during ICU stay were retrospectively reviewed. Only the first episode of ARF requiring RRT was taken into account. Patients with end-stage renal diseases requiring chronic dialysis were excluded. The study was approved by the ethical committee of the Institut Jules Bordet.

Acute renal failure was defined as a creatinine increase >2 mg/dl (176 μmol/l) in case of pre-existing normal value, or an increase >1 mg/dl from the basic value in case of chronic renal failure. Decision for CVVHDF was based on one (or more) of the following characteristics: bicarbonate level <10 mg/dl, metabolic acidosis (pH <7.30), uremia >33.2 mmol/l, kaliemia >6 mEq/dl, water overload, pericarditis secondary to ARF, uremic encephalitis, severe ionic abnormalities (hyponatremia, hyperphosphoremia), oligoanuria in presence of evolutive ARF.

The following clinical data were retrieved from the patient’s charts: gender, age, type of cancer, cancer phase (17) (diagnostic, curative, controllable but no more curable, pivotal or palliative), cancer status (18) (disease under induction therapy, complete remission without or on active anti-cancer treatment, partial remission, stable disease, progression), reason for ICU admission, anticancer treatments including bone marrow transplantation, potential nephrotoxic agents (iodine contrast agent, antibiotics, chemotherapy …), co-morbidities, ARF etiology, reason for CVVHDF, variables used for the computation of Simplified Acute Physiology Score II (SAPS II) (19).

The following data were collected at start of RRT: furosemide and vasopressive drugs administration, diuresis, blood pressure, heart and respiratory rates, temperature, biological variables, number of organ failures (including renal failure) at the time of ARF, the immunosuppression status according to the Organ Dysfunction and/or Infection (ODIN) score, the presence of an acute respiratory distress syndrome, and mechanical ventilation support.

We also recorded the main characteristics of RRT: duration, CVVHDF parameters, CVVHDF complications. To determine the final outcome of the patient, we assessed the renal function status (complete, partial or absence of return to initial creatinine values), mortality rates (during hospital and ICU stays), the date and cause of death, or last date known to be alive.

### Statistics and Data Analysis

Results are reported as medians and range. Prognostic factors for hospital mortality were tested in univariate analysis. Binary variables were compared by chi-square test or Fischer’s exact test. Non-parametric Mann–Whitney tests were used to compare continuous variables. Variables yielding *p* value less or equal to 0.2 in univariate analysis were entered into a multiple logistic regression. A Hosmer–Lemeshow test was performed to verify the goodness of fit. A *p* value <0.05 was considered statistically significant.

## Results

One-hundred and twelve patients were treated with RRT during the study period. One patient on chronic dialysis was excluded. Incomplete charts led to exclusion of a further 8 cases leaving 103 patients assessable for the final analysis.

The principal characteristics of the population are reported in Table 1. Renal failure primarily of renal origin, occurred in 83 patients, obstructive renal failure in 1 patient and both types combined in 19 patients. Etiologies of renal failure from renal origin were miscellaneous and detailed in Table 2. A combination of nephrotoxic insults was observed in 43 patients (42%). A number of potentially nephrotoxic drugs were implicated in the development of renal failure: antibiotics/antifungals/antivirals (*n* = 45), non-steroidal anti-inflammatory (*n* = 15), iodine contrast product (*n* = 15), chemotherapy agents (*n* = 12), furosemide (dose ≥500 mg/24 h) (*n* = 10), ciclosporine/sirolimus (*n* = 9), sartan/ACE inhibitor (*n* = 4), zoledronic acid/OKT3 (*n* = 2). A combination of nephrotoxics was found in 31 cases.

**Table 1:** **Characteristics at ICU admission of 103 patients with cancer with acute renal failure requiring renal replacement therapy**.

Sex (male/female)	69/34 (67%/33%)
Age (median/range)	62 years (19–87)
Solid malignancies (locoregional/metastatic)	68 (66%) (28/40)
Breast	7 (7%)
Lung	6 (6%)
Digestive tract	15 (15%)
Prostate/bladder/kidney	23 (22%)
Other	17 (17%)
Hematological malignancies	35 (34%)
Leukemia	10 (10%)
Lymphoma	14 (14%)
Multiple myeloma	9 (9%)
Myelodysplastic syndrome	2 (2%)
Stem cell transplantation before ICU admission (allo/auto)	18 (14/4) (17%)
Cancer phase	
Diagnostic	9 (9%)
Curative	41 (40%)
Controllable	53 (51%)
Cancer status	
Induction	25 (24%)
Diagnostic	9 (9%)
Remission (partial or complete)	35 (34%)
Stabilization	1 (1%)
Progression	33 (32%)
Causes of ICU admission	
Renal disease	58 (56%)
Shock/sepsis	20 (19%)
Metabolic disorder	9 (9%)
Respiratory failure	8 (8%)
Other	8 (8%)
Chemotherapy given before ICU admission	
Less than 15 days	35 (34%)
More than 15 days	27 (26%)
No	41 (40%)
Immunodepression	50 (49%)
Urinary tract infection	14 (14%)
Diabetes	14 (14%)
Baseline characteristics at the time of initiation of CVVHDF	
ARDS	25 (24%)
Mechanical ventilation	53 (51%)
Vasopressors	43 (42%)
Hematological failure	28 (27%)
>1 organ failure	65 (63%)
pH (median/range)	7.3 (7.02–7.52)
Natremia (mEq/l) (median/range)	138 (122–150)
Kaliemia (mEq/l) (median/range)	4.7 (2.9–8)
Creatininemia (mg/dl) (median/range)	3.76 (1.28–20.1)
Uremia (mg/dl) (median/range)	151 (49–492)
SAPS II (median/range)	56 (28–99)

*ICU, intensive care unit; SAPS, simplified acute physiology score; allo, allogenic; auto, autologous; ARDS, acute respiratory distress syndrome*.

**Table 2:** **Principal etiologies of acute renal failure in patients admitted in the ICU**.

	*n*	%^a^
Nephrotoxic drugs	48	47
Shock/sepsis	41	40
Acute tubular necrosis^b^	19	18
Cancer	10	10
Tumor lysis syndrome	8	8
MOF	4	4
Hepato-renal syndrome	4	4
Other	12	12
Unknown^c^	6	6

*^a^One patient could have had more than one etiological factor: a combination of at least two factors has been seen in 43 patients (42% of the cases)*.

*^b^Acute tubular necrosis except nephrotoxic drugs and shock/sepsis*.

*^c^No aetiology has been formally identified*.

*MOF, multiple organ failure*.

Criteria to initiate RRT were as follow: blood pH <7.3 (*n* = 28), (oligo)anuria (*n* = 17), uremia (*n* = 7), severe ionic abnormality (*n* = 6), water overload (*n* = 3), uremic encephalitis (*n* = 1), or a combination of these factors (*n* = 40). In one case, the precise indication for CVVHDF can not be retrospectively determined.

### CVVHDF Therapy Outcomes

Extra-renal replacement therapy was CVVHDF in 100 patients, CVVH (continuous veno-venous hemofiltration) and CVVD (continuous veno-venous dialysis) in 1 and 2 cases, respectively. Pre-dilution technique was applied in 54 cases, post-dilution in 35 cases and alternatively both in 12 cases. Median blood flow rate was 180 ml/min (range: 60–200). Median reinjection fluid rate was 28.6 ml/kg/h. The median cumulated overall hemofiltration duration was 34.5 h (range: 2–354 h). Complications of RRT were filter coagulation at least once (*n* = 32), hypotension (*n* = 3) and uncomplicated hemorrhage at the catheter insertion’s site (*n* = 3).

Complete or partial (persistent abnormal creatinine value not requiring further RRT) ARF resolution was observed in 27 and 18 patients, respectively. No resolution, defined as required RRT at the time of death (*n* = 49), transfer in another hospital without ARF resolution (*n* = 2), or need for chronic dialysis (*n* = 7), was found in 58 patients.

### Patient Survival Outcomes

Among patients alive at hospital discharge, complete, partial, or absence of ARF resolution was observed in 20, 9, and 9 patients, respectively. Seven patients (18% of the patients alive at the hospital discharge) needed chronic dialysis. Of them, 2 had a multiple myeloma and 5 patients presented with a loco-regional (*n* = 3) or metastatic (*n* = 2) solid tumor. Five of them had prior renal disease: polycystic kidney (*n* = 2), myelomatous kidney (*n* = 1), or a chronic renal failure not requiring dialysis (*n* = 2).

The median duration of ICU and hospital stays were 9 days (range: 1–124 days) and 22 days (range: 1–155 days). Fifty patients died in the ICU and a further 15 in the general ward after ICU discharge. Thirty-eight patients were discharged alive from the hospital. The ICU mortality rate was 49% and the hospital mortality rate was 63%. The main causes of hospital death were multiple organ failure (*n* = 21), septic shock (*n* = 16), shock from another origin (*n* = 7), cancer (*n* = 6), ARF (*n* = 2).

Univariate analysis of prognostic factors for hospital mortality is reported in Table 3. All significant variables and those with a *p*-value <0.2 in univariate analysis were included in a multivariate analysis. Two variables were statistically independent pejorative prognostic factors for hospital mortality in multivariate analysis: the number of organ failure [Odds ratio = 5.918; 95% confidence interval (CI) 2.184–16.038; *p* < 0.001] and hypoalbuminemia (Odds ratio = 3.341; 95% CI 1.229–9.077; *p* = 0.02).

**Table 3:** **Prognostic factors for hospital mortality – univariate analysis**.

		Non-survivors	Survivors	*p*-value
Hematological tumor		27 (77%)	8 (23%)	
Solid tumor		38 (55%)	30 (45%)	**0.034**
Age (median)		61 years	64 years	0.06
Number of organ failure				
1		10 (29%)	24 (71%)	**<0.00001**
>1		55 (80%)	14 (20%)	
Cancer status				
Progression		22 (67%)	11 (33%)	0.60
Other than progression		43 (61%)	27 (39%)	
Cancer phase				
Diagnostic		6 (67%)	3 (33%)	0.23
Curative		21 (51%)	20 (49%)	
Controllable		38 (72%)	15 (28%)	
Immunodepression	No	29 (55%)	24 (45%)	0.07
	Yes	36 (72%)	14 (28%)	
ARF origin	Renal	51 (61%)	32 (39%)	0.43
	Mixed	13 (68%)	6 (32%)	
Allo/auto graft	No	49 (58%)	36 (42%)	**0.01**
	Yes	16 (89%)	2 (11%)	
ARDS	No	47 (56%)	37 (44%)	**0.001**
	Yes	18 (95%)	1 (5%)	
Mechanical ventilation	No	22 (44%)	28 (56%)	**0.0001**
	Yes	43 (81%)	10 (19%)	
Vasopressor	No	30 (50%)	30 (50%)	**0.002**
	Yes	35 (81%)	8 (19%)	

		**Median (range)**	**Median (range)**	

SAPS II		57 (28–99)	50 (26–88)	**0.022**
Bilirubinemia (mg/dl)		1.4 (0.2–19)	0.5 (0.1–5.7)	**0.0002**
Creatininemia (mg/dl)		3.6 (1.3–18)	4.8 (1.3–20)	**0.02**
Uremia (mg/dl)		147 (55–492)	154 (49–485)	0.78
Natremia (mEq/l)		139 (122–150)	135 (128–148)	**0.01**
Kaliemia (mEq/l)		4.7 (3.2–8)	4.5 (2.9–7.8)	0.33
Phosphatemia (mEq/l)		6.2 (2.9–11.3)	6.1 (2.6–14.6)	0.80
Bicarbonate (mEq/l)		19 (6–32)	19 (3–27)	0.64
INR		1.45 (0.97–3.9)	1.33 (0.8–2.9)	0.15
Albuminemia (g/dl)		2.5 (0.7–4)	3.05 (1.7–5.1)	**0.0001**
Leukocytes (10^3^/mm^3^)		7.8 (0.02–39)	13 (0.05–50)	0.07

*SAPS, Simplified acute physiology score; INR, international normalized ratio; ARF, acute kidney injury; ARDS, acute respiratory distress syndrome*.

*Bold font highlight the significant variables in univariate analysis*.

At the time of analysis, 90 patients died, and 3 patients were lost to follow up. Median survival of patients discharged alive from hospital was 413 days; it was 157 days for patients on chronic dialysis but 441 days for those independent from dialysis (*p* = 0.34).

## Discussion

In this retrospective study including patients with cancer with ARF requiring RRT, two statistically significant independent prognostic factors for hospital mortality were identified. A higher risk of death was associated with increasing number of organ failures and with low albumin levels. Cancer characteristics and SAPS II score were not associated with outcome.

This study confirms that CVVHDF is a feasible and well-tolerated RRT in patients with cancer with ARF admitted to the ICU. Prognosis was comparable to previously published studies (Table 4). Detailed comparisons between studies are difficult due to different case-mix, indications and methods of RRT.

**Table 4:** **Summary of publications assessing renal replacement therapy in patients with cancer admitted into ICU**.

Reference	Population	*N*	RRT	Mortality	Prognostic factors for hospital mortality
**Mixed population (solid and hematological tumors, including bone marrow transplantation)**					
Berghmans et al. (15)	Solid: 50%	32	CVVHDF	ICU: 50%	Number of organ failure
	Hemato: 50%			Hospital: 53%	
	BMT: 28%				
Salahudeen et al. (5)	Solid: 38%	199	C-SLED	Day 30: 65%	SOFA score, pH, mean blood pressure
	Hemato: 62%				
	BMT: 18%				
**Mixed population (solid and hematological tumors, excluding bone marrow transplantation)**					
Maccariello et al. (13)	Solid: 73%	118	IRRT daily conventional	ICU: 70%	Number of organ failure
	Hemato: 27%		IRRT daily extended	Hospital: 78%	
			CRRT		
Darmon et al. (7)	Solid: 7%	94	CRRT	ICU: 43.6%	LOD score, late RRT (>24 h after ICU admission)
	Hemato: 78%		IRRT	Hospital: 51.1%	
	Other: 15%			6 months: 65.4%	
Soares et al. (14)	Solid: 75%	98	IRRT conventional	Hospital: 64–86%	–
	Hemato: 25%		IRRT extended		
			CRRT		
**Hematological tumors**					
Letourneau et al. (12)	BMT: 100%	14	CVVHDF	–	–
			IRRT		
Lanore et al. (11)	BMT: 11%	43		ICU: 72%	ARF secondary to sepsis, SAPS score, mechanical ventilation support
Benoit et al. (9)	BMT: 22.4%	50	IRRT	ICU: 79.6%	–
			CRRT	Hospital: 83.7%	
				6 months: 86%	

Cancer series found by PubMed search using the MESH terms “Acute kidney injury” and “Intensive care.”

*RRT, renal replacement therapy; CVVHDF, continuous venovenous hemodiafiltration; ICU, intensive care unit; C-SLED, sustained low efficiency dialysis in the continuous mode; CRRT, continuous renal replacement therapy; IRRT, intermittent renal replacement therapy; BMT, bone marrow transplantation; ARF, acute renal failure*.

As expected, higher mortality was observed when hematological malignancies are considered (9). Two prognostic factors for hospital mortality were highlighted that could be helpful in selecting patients for CVVHDF with better chance to be hospital discharged. The increased number of organ failures has yet been found associated with a pejorative prognosis as well in cancer and general populations (7, 13, 15, 16, 20, 21). On the opposite, the role of hypoalbuminemia was more controversial in general ICU (22–25) but was not extensively assessed in patients with cancer. A negative effect on mortality of hypoalbuminemia was shown in one study and only in univariate analysis (16). It must be emphasized that the cancer characteristics (status, phase or treatment) were not predictors of hospital mortality. As previously reported, hospital mortality was linked to acute physiological changes induced by the complications leading to ICU admission while cancer variables influenced significantly survival after hospital discharge (18, 26, 27).

In addition to the effectiveness of CVVHDF in patients with cancer, this RRT showed also adequate tolerance in this frail population, often immunocompromised due to cancer or its treatment. This observation was rarely reported in the literature. Other authors assessing C-SLED (sustained low efficiency dialysis in the continuous mode) for RRT, reported complication due to hypocalcemia induced by citrate administration for anticoagulation (16). In another publication concerning hematological malignancies, no bleeding was observed despite severe thrombopenia and a catheter infection was documented in 5% of the patients (11). Patients and physicians need to know that a substantial number of RRT will lead to permanent chronic replacement therapy, despite the documented effectiveness and tolerability of CVVHDF. In our study, 18% of the patients discharged alive from the hospital required a chronic dialysis. This was in the range between 14 and 24%, which is not different from rates observed in cancer patients (7, 13, 14) and in general ICU populations (28). It must be emphasized that in our study, five out of these seven patients presented with underlying renal diseases that could have contributed to the final renal status.

In the present study, we observed that half of the patients were exposed to a nephrotoxic drug that could have played a role in ARF development. Among them, anticancer drugs or immunosuppressive agents, the latter in case of bone marrow grafting, have been documented in 18% of the cases. A number of anticancer treatments are known for their nephrotoxicity with different pathophysiological mechanisms as cisplatin, nitrosourea derivatives, mitomycin, ifosfamide, or methotrexate without being exhaustive. Different preventive methods as adequate hydration (cisplatin) or urine alkalinization (high dose methotrexate) are recommended. Clinicians have to be aware of the poor prognosis of ARF when prescribing potentially nephrotoxic chemotherapy in patient with disturbed renal function and/or at risk for ARF. They also have to consider the risk of ARF when administering nephrotoxic agents after a nephrotoxic chemotherapy as iodine contrast product and/or aminoglycoside after cisplatin administration.

### Potential Bias and Limitations

Some bias related to retrospective studies must be discussed. As all the patients admitted to the ICU were prospectively recorded, a selection bias was unlikely. However, due to limited available data, some files have not been studied. The retrospective nature of our analysis could lead to missing data, which were minimal in our case. The number of patients in our study was limited but was in the same range as for the other publications. As for other unicentric studies, generalization of the results is limited. Nevertheless, our results and conclusion are comparable to previous series suggesting adequate robustness of the data (13, 29). Multiple definitions for ARF have been proposed. In this retrospective study, we decided, based on available data, to apply a simple definition of ARF (an increase of creatinine above 2 mg/dl). We did not use creatinine clearance or glomerular filtration rate because we were focusing on clinical settings where the clinicians decided performing RRT, most of the patients having high creatinine values and/or clinical signs of intolerance to ARF (acidosis, water overload …). Our data could deserve further prospective study in which a more precise definition of ARF should be used as RIFLE criteria or other clearance determination.

In conclusion, although ARF requiring RRT in patients with cancer admitted in ICU still has a poor prognosis, the use of CVVHDF is effective and tolerable in this immunocompromised population. Multiple organ failure and hypoalbuminemia have to be considered when deciding to introduce RRT in the treatment of this group of patients.

## Author Contributions

All the authors gave substantial contributions to the conception and design of the work, the acquisition, analysis, and interpretation of data for the work. They all revised the manuscript and approved the final version. They agree to be accountable for all aspects of the work in ensuring that questions related to the accuracy or integrity of any part of the work are appropriately investigated and resolved.

## Conflict of Interest Statement

The authors declare that the research was conducted in the absence of any commercial or financial relationships that could be construed as a potential conflict of interest.
